# Ihog and Boi are essential for Hedgehog signaling in *Drosophila*

**DOI:** 10.1186/1749-8104-5-28

**Published:** 2010-11-02

**Authors:** Darius Camp, Ko Currie, Alain Labbé, Donald J van Meyel, Frédéric Charron

**Affiliations:** 1 Molecular Biology of Neural Development, Institut de Recherches Cliniques de Montréal (IRCM), Montreal, QC, H2W 1R7, Canada; 2 Centre for Research in Neuroscience and the McGill University Health Centre Research Institute, 1650 Cedar Ave, Montreal, QC, H3G 1A4, Canada; 3 Division of Experimental Medicine, McGill University, Montreal, QC, Canada; 4 Department of Neurology and Neurosurgery, McGill University, Montreal, QC, Canada; 5 Department of Biology, McGill University, Montreal, QC, Canada; 6 Program in Neuroengineering, McGill University, Montreal, QC, Canada; 7 Department of Medicine, University of Montreal, Montreal, QC, Canada; 8 Department of Anatomy and Cell Biology, McGill University, Montreal, QC, Canada

## Abstract

**Background:**

The Hedgehog (Hh) signaling pathway is important for the development of a variety of tissues in both vertebrates and invertebrates. For example, in developing nervous systems Hh signaling is required for the normal differentiation of neural progenitors into mature neurons. The molecular signaling mechanism underlying the function of Hh is not fully understood. In *Drosophila*, Ihog (Interference hedgehog) and Boi (Brother of Ihog) are related transmembrane proteins of the immunoglobulin superfamily (IgSF) with orthologs in vertebrates. Members of this IgSF subfamily have been shown to bind Hh and promote pathway activation but their exact role in the Hh signaling pathway has remained elusive. To better understand this role *in vivo*, we generated loss-of-function mutations of the *ihog *and *boi *genes, and investigated their effects in developing eye and wing imaginal discs.

**Results:**

While mutation of either *ihog *or *boi *alone had no discernible effect on imaginal tissues, cells in the developing eye disc that were mutant for both *ihog *and *boi *failed to activate the Hh pathway, causing severe disruption of photoreceptor differentiation in the retina. In the anterior compartment of the developing wing disc, where different concentrations of the Hh morphogen elicit distinct cellular responses, cells mutant for both *ihog *and *boi *failed to activate responses at either high or low thresholds of Hh signaling. They also lost their affinity for neighboring cells and aberrantly sorted out from the anterior compartment of the wing disc into posterior territory. We found that *ihog *and *boi *are required for the accumulation of the essential Hh signaling mediator Smoothened (Smo) in Hh-responsive cells, providing evidence that Ihog and Boi act upstream of Smo in the Hh signaling pathway.

**Conclusions:**

The consequences of *boi;ihog *mutations for eye development, neural differentiation and wing patterning phenocopy those of *smo *mutations and uncover an essential role for Ihog and Boi in the Hh signaling pathway.

## Background

The Hedgehog (Hh) signaling pathway is essential for proper embryonic development, and aberrant Hh pathway activity is the cause of several human congenital defects and cancers [1-3]. In developing nervous systems, Hh is an important factor governing neural fate specification, neural precursor proliferation, and axon guidance [3-5]. For example, Hh signaling specifies neural cell fate identity in the developing neural tube of vertebrates [4], and is essential for the normal differentiation of photoreceptors in the *Drosophila *compound eye [6]. Despite its importance in these and many other developmental events in diverse species, the molecular signaling mechanism underlying the function of Hh is still not fully elucidated.

Hh is a secreted protein that elicits concentration-dependent effects [3,4]. Genetic and biochemical experiments have led to a model where Patched (Ptc), a 12-pass transmembrane protein, is involved in sensing the extracellular Hh concentration [2]. In the absence of Hh, Ptc maintains the 7-pass transmembrane protein Smoothened (Smo) in a repressed state. Under these conditions, the Cubitus interruptus (Ci) transcription factor is proteolytically cleaved and acts as a transcriptional repressor. Conversely, in the presence of Hh, Ptc-mediated repression of Smo is relieved and this leads to the stabilization of full-length Ci (Ci155), which activates transcription of Hh target genes.

Ihog (Interference hedgehog; CG9211) and Boi (Brother of Ihog; CG13756) are two related immunoglobulin superfamily (IgSF) members composed of four immunoglobulin-like (Ig) domains, two fibronectin type III (FN3) repeats, a transmembrane domain, and a cytoplasmic tail [7-9]. The first FN3 domain of Ihog is required and sufficient for direct binding to Hh [9,10]. In transcription reporter assays in cultured cells, modulation of Ihog and Boi levels affected the strength of responses to Hh [8,9]. Although Ptc plays a critical role in sensing the Hh morphogenic gradient, the identification of Ihog and Boi as proteins that bind to Hh and promote pathway activation raised questions about their exact role in the Hh signaling pathway. To better understand this role *in vivo*, we generated loss-of-function mutations of the *ihog *and *boi *genes in *Drosophila*. We have investigated the effects of these mutations in developing eye and wing imaginal discs, and found that Ihog and Boi are functionally redundant and are required for Hh signaling in these tissues. This work uncovers an essential role for Ihog and Boi in the Hh signaling pathway.

## Results

### Generation of *ihog *and *boi *mutants in *Drosophila*

**Figure 1:**
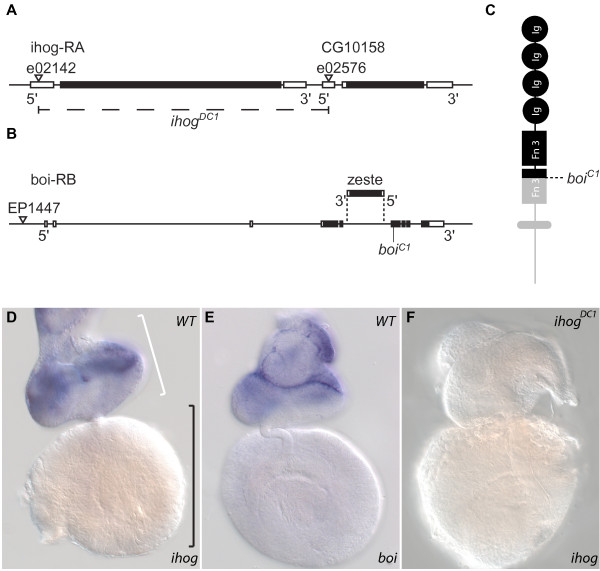
***ihog *and *boi *mutants**. **(A, B)**. Illustration of the intron-exon structure of the *ihog *and *boi *loci, with coding sequences shaded black. (A) The sites of *pBac *insertions used to create *ihog*^*DC1 *^are indicated (triangles), as is the extent of the 3,479-bp *ihog*^*DC1 *^deletion (dashed line). (B) An EP insertion (triangle) was used in a screen to isolate the nonsense *boi*^*C1 *^mutation. The location and orientation of the *zeste *gene within an intron of the *boi *gene are indicated. **(C) **Schematic diagram showing the protein structure common to both Ihog and Boi. The *boi*^*C1 *^mutation is predicted to truncate a significant portion of Boi (grey), including the second FN3 domain, transmembrane domain and cytoplasmic tail. **(D-F) **Whole mount *in situ *hybridization to third instar (L3) eye imaginal discs (white bracket) and connected brain hemispheres (black bracket). (D,E) In wild type (WT), *ihog *and *boi *transcripts are enriched in eye discs, where they appear to have overlapping expression. (F) *ihog *expression is not detectable in *ihog*^*DC1 *^null mutants.

**Figure 2:**
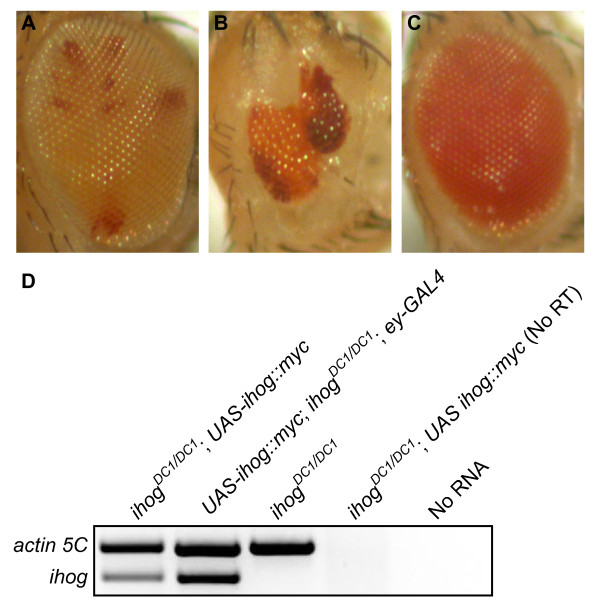
**Double mutations of *ihog *and *boi *severely disrupt eye development**. **(A) **Normal morphology of an *ihog*^*DC1 *^mosaic eye in a *boi*^*C1/+ *^heterozygote (genotype: *boi*^*C1 *^*/+; ihog*^*DC1*^, *FRT40A/FRT40A, l(2)CL-L1; ey-FLP/+*). Ommatidia composed of *ihog*^*DC1/DC1 *^homozygous cells are pigmented orange, while the dark red patches mark ommatidia with *ihog*^*DC1/+ *^heterozygous cells. **(B) **Eye morphology is severely disrupted in an *ihog*^*DC1 *^mosaic eye in a *boi*^*C1/- *^mutant (genotype: *boi*^*C1*^*/Y; ihog*^*DC1*^, *FRT40A/FRT40A, l(2)CL-L1; ey-FLP/+*). The eye is small, poorly shaped, and many ommatidia are missing. Some double mutant ommatidia appear to differentiate in these mosaics but have a roughened appearance, which is consistent with *smo *mutations. **(C) **Addition of Ihog rescues viability and eye morphology in a double mutant (genotype: *boi*^*C1*^*/Y; ihog*^*DC1/DC1*^*; UAS-ihog::myc/+*). **(D) **RT-PCR to demonstrate loss of *ihog *transcripts in *ihog*^*DC1/DC1 *^mutants, and to characterize a transgenic line with low-level, constitutive expression of *UAS-ihog::myc *in the absence of a Gal4 driver.

We generated an *ihog *null mutant (*ihog*^*DC1*^) that completely lacks the *ihog *coding sequence plus a portion of the 5' untranslated region of the adjacent gene CG10158 (Figure 1A; see Materials and methods). The absence of *ihog *mRNA transcripts in *ihog*^*DC1/DC1 *^mutants was confirmed by *in situ *hybridization (Figure 1F) and by reverse transcriptase PCR (RT-PCR; Figure 2D). *ihog*^*DC1/DC1 *^homozygotes or *ihog*^*DC1/Df *^hemizygotes were viable and fertile (Table 1). However, we observed semi-lethality when mutant progeny (*ihog*^*DC1/DC1 *^or *ihog*^*DC1/Df*^) but not heterozygous progeny (*ihog*^*DC1/+*^) were derived from *ihog*^*DC1/DC1 *^homozygous mothers (Table 1).

**Table 1:** Effect of *ihog *mutation on adult viability

	Expected	Observed
Cross: *ihog*^*DC1/+ *^females (f) × *ihog*^*Df/+ *^males (m)		
F1 (n = 208)		
*ihog*^*+/+*^	25%	20%^a^
*ihog*^*DC1/+*^	25%	21%^a^
*ihog*^*Df/+*^	25%	35%^a^
*ihog*^*DC1/Df*^	25%	24%^a^
		
Cross: *ihog*^*DC1/DC1 *^(f) × *ihog*^*Df/+ *^(m)		
F1 (n = 125)		
*ihog*^*DC1/+*^	50%	90%^b^
*ihog*^*DC1/Df*^	50%	10%^b^
		
Cross: *ihog*^*DC1/DC1 *^(f) × *ihog*^*DC1/+*^; *UAS ihog::myc/+ *(m)		
F1 (n = 73)		
*ihog*^*DC1/DC1*^; *UAS ihog::myc/+*	25%	29%^b^
*ihog*^*DC1/DC1*^	25%	5%^b^
*ihog*^*DC1/+*^; *UAS ihog::myc*	25%	33%^b^
*ihog*^*DC1/+*^	25%	33%^b^

^a^The observed segregation ratio is not statistically different from expected (chi-square test: *P *> 0.05). ^b^The observed segregation ratio is statistically different from expected (chi-square test: *P *< 0.05).

We characterized a *UAS-ihog:myc *transgenic line with basal levels of expression in the absence of a Gal4 driver (Figure 2D). Eclosion and adult viability were fully rescued in the presence of *UAS-ihog::myc *(Table 1), demonstrating that death at pupal stages was caused by loss of Ihog function and not CG10158. Together, the data indicate that zygotic Ihog is important for adult viability only in the absence of maternal Ihog.

The viability and lack of overt Hh-like phenotypes in *ihog*^*DC1 *^mutants prompted us to also investigate Boi, since it behaves similarly to Ihog in Hh transcription reporter assays *in vitro *[9]. We used chemical mutagenesis to generate a *boi *mutation (*boi*^*C1*^) in a genetic screen (Figure 1B; Additional file 1; see Materials and methods). *boi*^*C1 *^causes a premature stop codon at amino acid Trp626 (Figure 1B). This mutation is predicted to truncate the Boi protein within the second FN3 domain, and fails to encode the transmembrane domain and cytoplasmic tail (Figure 1C). Therefore, the *boi*^*C1 *^mutation is predicted to severely disrupt the properties of Boi. The *boi *gene is situated on the X chromosome and therefore males are *boi*^*C1/- *^hemizygotes. Similar to *ihog, boi*^*C1/C1 *^female flies and *boi*^*C1/- *^male flies were viable and fertile, but unlike *ihog *there was no maternal effect.

Thus, zygotic mutants for either *ihog*^*DC1 *^or *boi*^*C1 *^were viable and exhibited no overt phenotype reminiscent of mutations of components of the Hh signaling pathway. However, larvae that were double mutants for *ihog*^*DC1 *^and *boi*^*C1 *^died 24 to 48 hours after hatching, and never reached third instar (L3), suggesting that *ihog *and *boi *might act redundantly. In support of this idea, viability of double mutants was rescued in the presence of *UAS-ihog::myc*, suggesting that Ihog could supplant Boi function in these flies (Figure 2C).

### Effects of *ihog *and *boi *mutations in the developing visual system

In the developing *Drosophila *retina, neuronal differentiation within the eye imaginal disc proceeds stepwise in the wake of a constriction in the disc epithelium called the morphogenetic furrow (MF) [11]. Hh signaling is required for the initiation of the MF at the posterior margin of the disc, and for its wave-like progression across the disc that marks the boundary between undifferentiated anterior cells and differentiating posterior cells [6]. Previous studies have demonstrated that clones of cells in the developing eye disc that lack the essential Hh mediator Smo retard MF progression and exhibit a cell-autonomous delay of differentiation into photoreceptors (R-cells) [12,13].

To begin to investigate whether *ihog *and *boi *could play a role in the developing visual system, we studied their expression in wild-type L3 larvae using *in situ *hybridization (Figure 1D, E). Transcripts for both genes were observed throughout the developing eye disc, but were enriched in anterior regions characterized by undifferentiated cells compared to the posterior regions where there is ongoing differentiation of retinal cell types. Transcripts for neither *ihog *nor *boi *were detected in the optic lobe and other regions of the brain at this stage. We confirmed the complete loss of *ihog *mRNA in *ihog*^*DC1 *^mutants (Figure 1F) and thereby demonstrated the specificity of the *ihog *probe. We could not do likewise with the *boi *probe, since it was predicted to hybridize with transcripts from the *boi*^*C1 *^nonsense mutation. However, the *boi *probe is unlikely to cross-hybridize with *ihog *transcripts as the *ihog *probe does not detect *boi *transcripts in *ihog*^*DC1/DC1 *^mutants. Together, these data revealed overlapping expression of *ihog *and *boi *in the developing eye disc, supporting the idea that they might act redundantly in this context.

To test this directly, we needed to generate mosaic animals because, as noted above, double mutant larvae died prior to L3. In *boi*^*C1/- *^hemizygotes, we used FLP-mediated mitotic recombination to render the majority of cells in the developing eye discs also mutant for *ihog*^*DC1/DC1 *^[14]. Eye development was severely compromised in these mosaic animals (Figure 2B), leading to structural defects reminiscent of those found in *smo *and *hh *mosaics [15-17]. Control mosaics in which *ihog*^*DC1/DC1 *^eye discs were generated in *boi*^*C1/+ *^heterozygotes had normal eyes (Figure 2A). Additional controls with normal eyes were *boi*^*C1/-*^; *ihog*^*DC1/+ *^males (not shown) and rescued double mutants (genotype: *boi*^*C1/-*^; *ihog*^*DC1/DC1*^; *UAS-ihog::myc*/+; Figure 2C). These results indicated that Ihog and Boi are together required for the proper development of the *Drosophila *eye.

### Ihog and Boi are required for Hh signaling and neuronal differentiation in the eye imaginal disc

**Figure 3:**
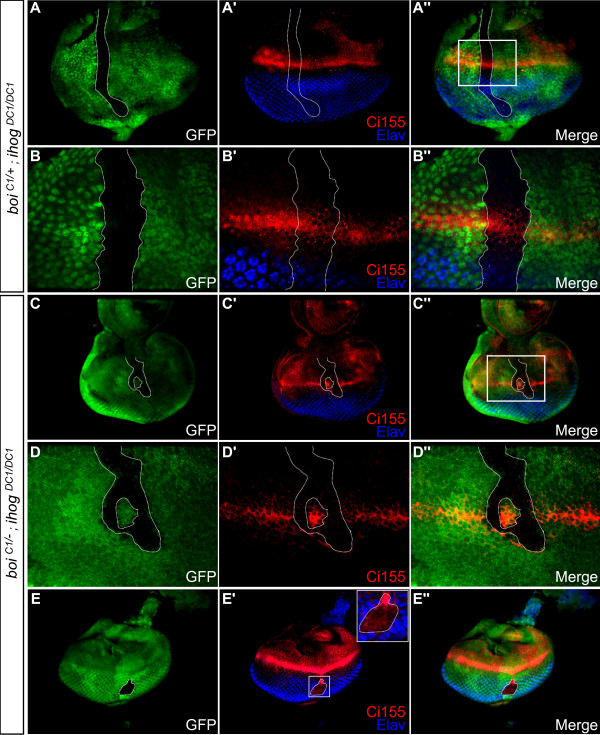
**Cells mutant for both *ihog *and *boi *do not activate the Hh pathway, disrupting R-cell differentiation in the developing eye disc**. **(A-B'') **Control in which an *ihog*^*DC1/DC1 *^clone (green fluorescent protein (GFP)-negative cells marked by dotted line) was generated in an eye disc of a *boi*^*C1/+ *^heterozygote. (A-A'') Low magnification view of entire disc. (B-B'') High magnification view of boxed area in (A''). Control clones show normal accumulation of cytoplasmic Ci155 (red) near the MF, and normal expression of Elav (blue) among differentiating R-cells posterior to the MF. **(C-E'') **An *ihog*^*DC1/DC1 *^clone (dotted line) in a *boi*^*C1/- *^mutant. (C-C'') Low magnification view. (D-D'') Higher magnification view of boxed area in (C''). Ci155 expression (red) is undetectable in cells mutant for both *ihog *and *boi *(GFP-negative, dotted line). (E-E'') In a double mutant clone situated well posterior to the MF, the absence of Elav and the ectopic expression of Ci155 indicate a delay of R-cell differentiation. Anterior is at the top in all panels.

To determine whether the disruption of eye development that we observed was due to perturbation of the Hh signaling pathway, we first examined the distribution of Ci155, a Gli-related zinc-finger protein that regulates transcription of Hh-responsive genes. Hh signaling inhibits proteolytic processing of Ci155 to the truncated repressor form Ci75 [3,18]. Using an antibody that detects Ci155 but not Ci75, one can monitor Hh signaling in cells that accumulate high levels of Ci155 [19]. In eye discs of L3 larvae, Ci155 normally accumulates to high levels just anterior to the advancing MF, marking Hh signaling that is required for MF progression [12,20,21]. In control experiments, we generated *ihog*^*DC1/DC1 *^clones in *boi*^*C1/+ *^heterozygotes and found no effect on Ci155 accumulation just anterior to the MF (eight of eight clones; Figure 3A-B''). In contrast, *ihog*^*DC1/DC1 *^clones in *boi*^*C1/- *^mutants had no detectable Ci155 expression at the MF (11 of 11 clones; Figure 3C-D''), indicating a loss of Hh signal transduction in double mutant cells.

Like *smo *mutant clones [12,15], we found that *boi;ihog *double mutant clones failed to express Elav, a neuron-specific marker for R-cells in the eye disc (green fluorescent protein (GFP)-negative in Figure 3E-E''). In controls, cells posterior to the MF that were mutant for only *ihog *(GFP-negative in Figure 3A, B) or only *boi *(GFP-positive in Figure 3E) expressed Elav normally, indicating that simultaneous loss of both family members is required to affect R-cell differentiation.

Elevated Ci155 levels drop sharply in cells posterior to the MF in a Cullin-3-dependent proteolytic process associated with the onset of R-cell differentiation [12,15]. As in the case of *smo *mutant clones [12,15], the absence of R-cells in clones lacking *ihog *and *boi *is accompanied by ectopic accumulation of Ci155 in clones located posterior to the MF (Figure 3E-E''). Taken together, our data suggest that Ihog and Boi are functionally redundant and, like Smo, are essential for the differentiation of R-cells in response to Hh.

### Ihog and Boi are required cell-autonomously for both high- and low-threshold responses to Hh pathway activation

**Figure 4:**
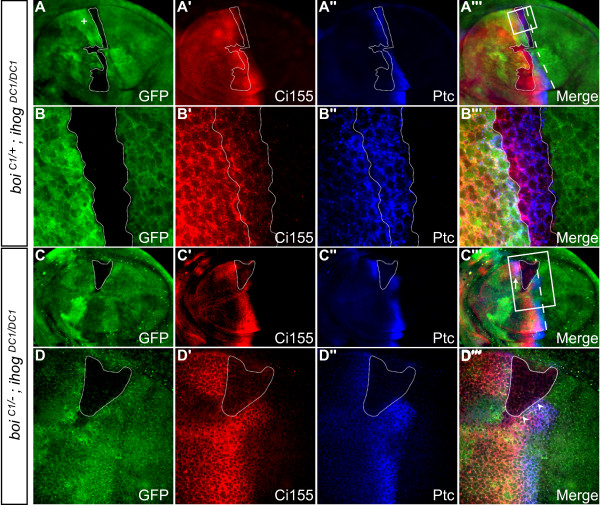
***ihog *and *boi *are required for high- and low-threshold responses to Hh pathway activation**. All panels show third instar wing imaginal discs with anterior to the left. **(A-B''') **Control showing large GFP-negative *ihog*^*DC1/DC1 *^clone (outlined with dotted line) in a *boi*^*C1/+ *^heterozygote. The adjacent twin-spot is marked by the high level of GFP staining (the plus sign in (A)). (A-A''') Low magnification view. The white dashed line in (A''') marks the normal position of the anterior-posterior boundary. (B-B''') Higher magnification view of boxed area in (A'''). Ptc (blue) is a high-threshold Hh target that is normally activated in anterior cells immediately adjacent to the anterior-posterior compartment boundary, while Ci155 (red) accumulates in cells positioned more anteriorly in response to lower levels of Hh signaling. In this large anterior control clone, there is normal expression of Ptc and Ci155 and no segregation into posterior territory. **(C-D''') **An *ihog*^*DC1/DC1 *^clone (dotted line) in a *boi*^*C1/- *^mutant. (C-C''') Low magnification view. White dashed line in (C''') marks the normal position of the anterior-posterior boundary. (D-D''') Higher magnification view of boxed area in (C'''). Clones lacking both *ihog *and *boi *were unable to express Ptc or accumulate high levels of Ci155 (GFP-negative, dotted line). High levels of Ptc and Ci155 in cells immediately adjacent to clones (arrowheads in (D''')) indicate that Ihog and Boi are required cell-autonomously in Hh responding cells, and expression of these markers immediately anterior to the clone (arrow in (C''')) indicates that loss of Ihog and Boi fails to sequester Hh activity in the clone.

To determine whether Ihog and Boi also function in Hh signaling elsewhere, and to explore whether they mediate high-threshold and low-threshold responses to a Hh morphogen gradient *in vivo*, we examined their role in wing development. Subdivision of the developing imaginal wing disc into anterior and posterior compartments involves the posterior-specific expression of the selector gene Engrailed [22], which programs posterior cells to produce and secrete Hh, and simultaneously prevents their response to Hh by blocking expression of Ci [23,24]. Therefore, only anterior cells limited to a broad stripe along the anterior/posterior compartment boundary can respond to Hh, and they do so by upregulating the expression of Hh target genes in a manner that depends on their proximity to the boundary and therefore the concentration of Hh to which they are exposed [25]. Cells immediately adjacent to the compartment boundary respond to high levels of Hh by expressing Ptc, a direct transcriptional target of Hh signaling [26]. Cells positioned more anteriorly respond to lower levels of Hh marked by high Ci155 accumulation, though all anterior cells have low baseline levels of Ci155. In control experiments, levels of Ptc and Ci155 were unaffected in *ihog*^*DC1/DC1 *^mutants (not shown), in *boi*^*C1/- *^hemizygous cells (GFP-positive in Figure 4C, D) and in *ihog*^*DC1/DC1 *^clones in *boi*^*C1/+ *^heterozygotes (12 of 12 clones; GFP-negative in Figure 4A, B).

In contrast, cells lacking both *ihog *and *boi *in anterior clones situated near the compartment boundary did not express Ptc and did not accumulate high levels of Ci155 (12 of 12 clones; Figure 4C-D'''), similar to *smo *clones [27]. Ci155 normally accumulates to high levels even at low concentrations of Hh [28,29], and so the failure to elicit this low-threshold response indicates that removal of *ihog *and *boi *results in complete loss of Hh pathway activation. Importantly, while cells lacking both *ihog *and *boi *lost the ability to respond to Hh, cells immediately adjacent to these clones expressed Ptc and accumulated Ci155 normally (arrowheads in Figure 4D'''), indicating that Ihog and Boi are required cell-autonomously in Hh responding cells.

### Ihog and Boi sequester Hh activity

In response to Hh, upregulation of Ptc levels in anterior cells near the compartment boundary reduces the anterior range of Hh activity [27]. This finding has been fundamental to the prevailing model that Ptc is a Hh receptor that can directly bind and sequester Hh protein. An alternative hypothesis is that Ptc interacts indirectly with Hh, perhaps by tethering another protein that directly binds Hh [27]. Since Ihog and Boi bind directly to Hh *in vitro *[9], and since Ptc and Ihog have been shown to synergize to increase binding of Hh to cultured cells [9], we wondered whether Ihog and Boi could likewise limit the range of Hh activity within the anterior compartment of the wing disc. Therefore, we studied large anterior clones that spanned the usual Ptc expression domain, and examined Ptc and Ci levels in cells immediately anterior to these clones. In cells that were immediately anterior to control *ihog*^*DC1/DC1 *^clones in *boi*^*C1/+ *^heterozygotes, there was no evidence for Hh pathway activation. In contrast, high levels of both Ptc and Ci155 were observed in cells immediately anterior to clones lacking Ihog and Boi (Figure 4C-D'''), indicating that the loss of both Ihog and Boi fails to restrict the movement of Hh through the clone.

### Ihog and Boi act upstream of Smo

**Figure 5:**
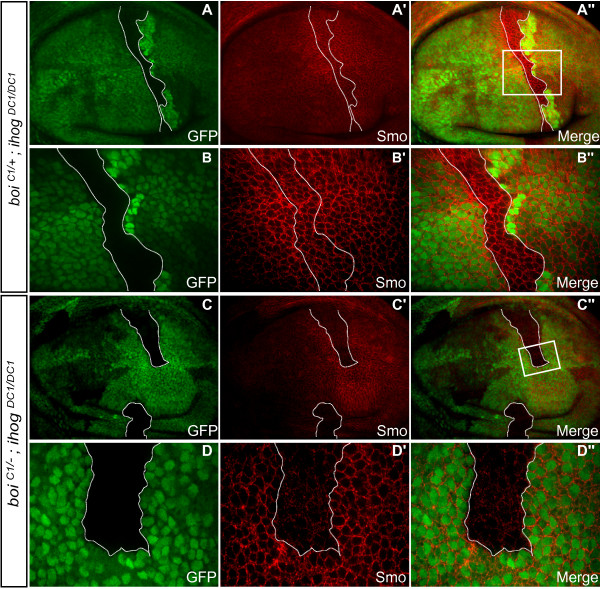
***ihog *and *boi *are required for Smo accumulation**. All panels show third instar wing imaginal discs with anterior to the left. **(A-B'') **In controls, *ihog*^*DC1/DC1 *^clones (dotted line) were made in a *boi*^*C1/+ *^heterogygote. (A-A'') Low magnification view. (B-B'') Higher magnification view of boxed area in (A''). Anti-Smo immunoreactivity (red) is normally enriched in the posterior versus anterior compartment, but highest accumulation of Smo occurs in Hh-responsive anterior cells immediately adjacent to the compartment boundary. Within the control clone indicated, Smo levels were unaffected. **(C-D'') **An *ihog*^*DC1/DC1 *^clone (dotted line) in a *boi*^*C1/- *^mutant. (C-C'') Low magnification view. (D-D'') Higher magnification view of boxed area in (C''). Within clones lacking both *ihog *and *boi*, Smo staining levels were markedly reduced.

Activation of the Hh signaling pathway leads to increased accumulation of Smo protein at the surface of cells near the compartment boundary of the wing disc [30]. This accumulation of Smo at the cell surface is required for its signaling activity, and therefore marks Smo activation by Hh [30-33]. Thus, to test whether Ihog and Boi could function upstream of Smo, we examined Smo accumulation in *ihog;boi *mutant clones. While control clones showed normal Smo accumulation (eight of eight clones; Figure 5A-B''), cells within *ihog;boi *mutant clones had Smo protein levels that were markedly reduced (seven of seven clones; Figure 5C-D''). Thus, Ihog and Boi are required for the Hh-dependent stabilization of Smo, and these results provide evidence that Ihog and Boi act upstream of Smo in the Hh signaling pathway.

### Ihog and Boi are required for compartment-specific cell affinity

**Figure 6:**
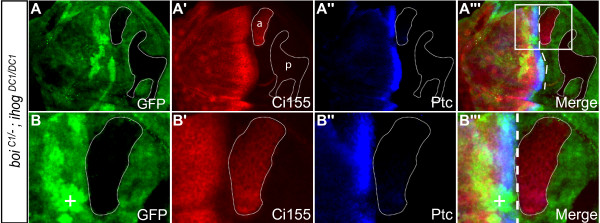
***ihog *and *boi *are required to maintain the affinity boundary between anterior and posterior compartments**. All panels show third instar wing imaginal discs with anterior to the left. **(A-B''') ***ihog*^*DC1/DC1 *^clones (dotted line) in a *boi*^*C1/- *^mutant. (A-A''') Low magnification view. A clone of anterior origin (labelled a in (A')) is identified by the expression of basal levels of Ci155. A clone of posterior origin (labelled p in (A')) does not express Ci155. (B-B''') Higher magnification view of boxed area in (A'''). The anterior-derived double mutant clone (dotted line) is found in posterior territory. The adjacent twin-spot is marked by the high level of GFP staining (labelled with the plus sign in (B,B''')). The white dashed line in (A''',B''') marks the normal position of the anterior-posterior boundary.

Cells of the wing imaginal disc normally do not cross the boundary between the anterior and posterior compartments, and implementation of this boundary is thought to involve mechanisms that control compartment-specific cell affinity and adhesion [34]. Clonal analysis has demonstrated that Hh signaling is required to maintain cell affinities in the anterior compartment. For example, if anterior cells that are adjacent to the boundary are mutant for *smo *or *ci *[35-37], they sort out from other anterior cells and segregate into posterior territory. To determine if Ihog and Boi are involved in maintaining compartment-specific cell affinities, we examined the segregation behavior of clones near the anterior-posterior compartment boundary. To do this, clones of anterior origin were unambiguously identified if they expressed baseline levels of Ci, and if an adjacent wild-type sister clone, the twin-spot, was situated in the anterior compartment since, by definition, the mutant clone and twin-spot must arise from the same compartment. In controls (*ihog*^*DC1/DC1 *^clones in *boi*^*C1/+ *^heterozygotes), anterior-derived clones exclusively occupied anterior territory and defined a straight border with posterior cells in the normal position of the boundary (14 of 14 clones; Figure 4A-B'''). In contrast, double mutant clones (*ihog*^*DC1/DC1 *^clones in *boi*^*C1/- *^hemizygotes) often straddled the boundary and formed tight borders with both anterior and posterior cells (8 of 16 clones; Figure 6). This modified segregation behavior indicates that anterior cells lacking both Ihog and Boi sort out from the anterior compartment into posterior territory. These clones do not readily integrate into posterior territory because they do not possess affinity for posterior cells conferred by expression of Engrailed [35]. Consistent with the lack of a known role for Hh signaling in the posterior compartment, double mutant posterior clones respected the compartment boundary and did not sort into anterior territory (11 of 11 clones; Figure 6A'). Together, these results indicate that, like Smo or Ci, Ihog and Boi are required to maintain cell affinities in the anterior compartment.

## Discussion

### Ihog and Boi are essential components of the Hh pathway

Using genetic approaches in *Drosophila*, we show that *ihog *and *boi *act redundantly and are required for viability. Mutation of *ihog *and *boi *in the developing eye disc prevents Hh signaling and causes severe disruption of photoreceptor differentiation. Similarly, mutation of *ihog *and *boi *in the developing wing disc completely abrogates Hh signaling in a cell-autonomous manner. All these phenotypic effects are identical to mutations of the essential Hh signaling mediator *smo *and thus indicate that the transmembrane proteins Ihog and Boi are absolutely required for the Hh signaling pathway. We surmise that the essential role for *ihog *and *boi *in Hh signaling was not discovered previously because they are redundant with one another, and therefore refractory to conventional forward genetic screens in *Drosophila*.

A previous study of the lethal mutation *ihog*^*KG *^reported defects that mimicked those caused by mutations of the Hh signaling pathway [9]. That *ihog*^*KG *^disrupts gene(s) in addition to *ihog *is indicated by the fact that zygotic mutation of *ihog*^*KG *^alone is lethal, while the null allele *ihog*^*DC1 *^is not. *ihog*^*KG *^was reported to disrupt Ptc expression in the wing disc, and the patterning of wing veins [9], but we did not observe these effects with *ihog*^*DC1 *^null mutations (Figure 4A'', B''). In addition, germline clones of *ihog*^*KG *^were found to disrupt patterning of the embryonic cuticle [9]. Therefore, it was unexpected that our maternal/zygotic *ihog *^*DC1 *^null mutants survived at least to pupal stages, and that there were no overt Hh-like phenotypes in the occasional flies that managed to escape the lethal phase and reach adulthood. Indeed, such flies looked remarkably normal. Perhaps the phenotypes observed in *ihog*^*KG *^are rare, or due to the disrupted function of the neighboring gene CG10158 or another gene in addition to *ihog*.

In *Drosophila*, genetic experiments have suggested that Ptc is the Hh receptor [38] and subsequent work has shown Ptc co-localization with Hh in S2 cells [39]. This has led to the model that Ptc binds Hh, though experiments have yet to demonstrate direct physical contact. In vertebrates, expression of the Ptc ortholog Ptc1 in cells promoted Sonic hedgehog (Shh) binding, suggesting that Ptc1 is a receptor for Shh [40,41]. However, as noted by the authors in those studies, Shh could use additional receptors and Ptc could affect their ability to bind to Shh. Addressing the important question of whether Ptc/Ptc1 bind directly to Hh/Shh is likely to require purified proteins in cell-free systems, a challenge for transmembrane proteins with complex topologies like Ptc. Interestingly, Ihog and Boi have been shown to bind directly to Hh [9,10]. Here we found that clones mutant for both *ihog *and *boi *phenocopied the effects of *smo *mutations for Hh signaling. Thus, our data demonstrate that Ihog and Boi are essential components of the Hh pathway. Coupled with our finding that Ihog and Boi act upstream of Smo and sequester Hh activity, this raises the possibility that Ihog and Boi are Hh-binding components of the Hh receptor complex. While preparing this manuscript, another research group came to a similar conclusion [42]. Using biochemical experiments, they further suggest that Ihog/Boi, together with Ptc and Hh, form a complex and that the presence of Ihog/Boi in this complex is essential to allow Ptc to bind to Hh. However, since purified proteins were not used in these experiments, it remains to be determined whether the interaction between Hh and Ptc is direct. Alternatively, Ptc could affect the ability of an additional protein (perhaps Ihog or Boi) to recruit Hh. Thus, the question as to whether Ptc binds directly to Hh, in the absence or the presence of Ihog/Boi, remains to be elucidated. Additional experiments will be required to understand the exact contributions of Ihog, Boi and Ptc to the reception and transduction of the Hh signal. Nonetheless, our experiments and those of Zheng and colleagues identify Ihog and Boi as essential components of a Hh receptor complex.

In addition to Ihog and Boi, other membrane-tethered Hh-binding proteins, such as the glypican family members, have been identified [8,43-45]. Although it is possible that other developing tissues may use different Hh-binding molecules as sensors for Hh, our data indicate that Ihog and Boi are critical to elicit Hh signaling and that, at least in developing imaginal tissues, no other molecule can compensate for loss of Ihog and Boi.

### Ihog and Boi: transmembrane IgSF proteins with potential to mediate cell affinity

In addition to the role of Ihog and Boi in wing cell fate specification (Zheng et al. [42] and this study), we show for the first time that Ihog and Boi are required for maintenance of the antero-posterior boundary of the wing disc. Formation of this boundary is thought to involve cell-surface recognition molecules responsible for affinity among anterior cells. Since *ci *mutant clones lose affinity for the anterior compartment [37], it is likely that such adhesion molecules are transcriptionally regulated by the Hh pathway. Despite broad screening efforts to identify these molecules [46], they remain unidentified. While the loss of cell affinity in *boi;ihog *double mutant clones is likely a consequence of loss of Hh signaling, it is an intriguing possibility that Ihog and/or Boi could also be Ci-regulated recognition molecules promoting the differential compartment-specific cell affinity. Consistent with this hypothesis, the vertebrate orthologs of Ihog and Boi, called Cdon (Cell adhesion molecule-related/down-regulated by oncogenes) and Boc (Brother of Cdon), can interact with each other and form higher-order cell-surface complexes with the well-characterized adhesion molecules N- and M-cadherin [7,47]. These complexes mediate cell-cell interactions between muscle precursor cells and are enriched at sites of contact between myoblasts. Similar to Cdon and Boc, Ihog and Boi could mediate cell-cell interactions, and might play a direct role in compartment-specific cell affinity.

### Ihog and Boi and their vertebrate orthologs in nervous system development

Although Cdon and Boc have also been shown to play a role in Hh signaling in vertebrates [9,48-50], their absolute requirement for Hh signaling has not been demonstrated, and mouse embryos mutant for both *cdon *and *boc *do not display a *smo*-like phenotype (our unpublished data). Additionally, Hh binds to Ihog/Boi in a manner that is different from Shh binding to Cdon and Boc [10,51], suggesting that a direct extrapolation of our work in flies to Cdon and Boc could be subject to added complexities.

Nevertheless, there are enough interesting parallels between Ihog and Boi and their vertebrate orthologs in the developing nervous system to warrant further investigation. We have shown here that Ihog and Boi are essential for the differentiation of R-cells in the developing fly visual system. Interestingly, overexpression studies in chick have shown that Boc and Cdon promote Shh-induced differentiation in the neural tube and that Cdon is required for Shh-dependent cell fate specification in the neural tube [48,50]. Furthermore, Boc has been shown to be essential for axon guidance through the activation of a non-canonical, transcription-independent Shh signaling pathway mediated by the activity of Src-family kinases [49,52,53]. It will be interesting to determine whether Ihog and Boi also function in axon guidance and whether they may contribute to non-canonical Hh signaling in *Drosophila*.

## Conclusions

We conclude that the transmembrane proteins Ihog and Boi are essential components of the Hh signaling pathway. Ihog and Boi act upstream of Smo and sequester Hh activity, raising the possibility that Ihog and Boi are Hh-binding components of the Hh receptor complex. These findings advance our understanding of the molecular signaling mechanism underlying the function of Hh.

## Materials and methods

### Fly stocks

*ihog*^*DC1 *^was generated by Flippase (FLP)-mediated recombination between Flippase recognition target (FRT) sites in pBac{RB}CG10158^e02576 ^and pBac{RB}iHog^e02142 ^[54,55]. After out-crossing to *w*^*1118*^, the resulting chimeric pBac element was excised, and the predicted deletion of 3,479 bp was confirmed by DNA sequencing and RT-PCR (Figure 2D). A deficiency stock that uncovers the *ihog *locus (Df(2L)Exel7029) was also used, and is designated *ihog*^*Df *^herein.

*boi*^*C1 *^was identified in a screen for mutations that suppress ectopic wing veins caused by *ap*^*GAL4*^-driven expression of P{EP}EP1447, a UAS-based P-element situated upstream of the *boi *gene (Figure S1A). Briefly, starved EP1447 male flies were exposed to 25 mM ethylmethanesulfonate (EMS) overnight, then crossed to *ap*^*GAL4 *^virgin females. *boi*^*C1 *^was identified among approximately 15,000 adult progeny screened for suppression of ectopic wing veins. *boi*^*C1 *^proved to be a viable mutation, and so PCR and DNA sequencing were used to compare the *boi *coding region in *boi*^*C1 *^homozygotes with the parental line EP1447. An induced G-to-A transition was found in *boi*^*C1*^, resulting in a nonsense mutation at Trp626.

To create *UAS-ihog::myc*, the entire coding sequence of *ihog *(minus stop codon) was PCR-amplified from an *ihog *cDNA (GH03927), and cloned into pCR2.1-TOPO (Invitrogen, Carlsbad, CA, USA). This fragment was excised and cloned into p5MT2Stp (BamHI/SalI) to add five copies of the myc epitope to the carboxyl terminus. *UAS- ihog::myc *was generated by KpnI/NotI excision and cloning into pUASt. Transgenic flies were generated in a w- background by standard microinjection procedures (BestGene Inc., San Diego, CA, USA).

### Mosaic analysis

Mosaic adult eyes were obtained by crossing *boi*^*C1/C1*^*; ihog*^*DC1*^, *FRT40A/+ *females to *FRT40A, l(2)CL-L*^*1*^*; ey-FLP *males [14]. Wing and eye disc clones marked by the absence of GFP were generated by crossing *boi*^*C1/C1*^*; ihog*^*DC1*^, *FRT40A/+ *females to *Ubi-GFP, FRT40A; hs-FLP *males. In this way, *ihog*^*DC1/DC1 *^clones would be generated in *boi*^*C1/+ *^heterozygotes (female progeny) or *boi*^*C1/- *^hemizygotes (male progeny). Embryos were collected for 16 hours, raised for 24 hours (25°C), then the larvae were heat shocked at 38°C for 1 hour and further raised (25°C) through L3.

### Immunohistochemistry

Wandering L3 larvae were dissected and fixed according to standard procedures, just prior to pupation. Monoclonal antibodies obtained from the Developmental Studies Hybridoma Bank included: mouse anti-Ptc (dilution 1:50), mouse anti-Smo (1:50) and mouse anti-Elav (1:2,000). Other antibodies used were rabbit anti-GFP (1:1,000; Molecular Probes, Eugene, OR, USA) and rat anti-Ci155 (1:2,000) [19]. Secondary antibodies were: Alexa Fluor 488 conjugated goat anti-rabbit (1:300); Alexa Fluor 647 conjugated goat anti-mouse (1:300); and Alexa Fluor 588 conjugated goat anti-rat (1:300), all from Molecular Probes.

### *In situ *hybridization

A digoxigenin (Dig)-labeling kit (Roche, Indianapolis, IN, USA) was used to synthesize cRNA probes from a BglII-digested *ihog *cDNA (GH03927) and an EcoRI-digested *boi *cDNA (SD07678), using T7 and Sp6 RNA polymerases, respectively. Prepared L3 larvae were hybridized with probes overnight at 55°C using standard procedures, and visualized using anti-Dig-AP (1:1,000; Roche).

### RT-PCR

RT-PCR was performed on total RNA isolated from adult flies. The *ihog *primer set was designed to amplify a band of 411 bp from the *UAS-ihog::myc *transgene, but not *ihog*^*DC1/DC1 *^mutants (5'-CCCTGAGCAAGTGTGGAGAT-3'; 5'-CTCTAGGCGAGTACCGATGC-3'). An *Actin5C *fragment of 586 bp was co-amplified as a control (5'-GAGCGCGGTTACTCTTTCAC-3'; 5'-ATCCCGATCCTGATCCTCTT-3').

## Abbreviations

BOC: Brother of Cdon; BOI: Brother of Ihog; BP: base pair; CDON: Cell adhesion molecule-related/down-regulated by oncogenes; CI: Cubitus interruptus; DIG: digoxigenin; FN3: fibronectin type III; GFP: green fluorescent protein; HH: Hedgehog; IG: immunoglobulin-like; IGSF: immunoglobulin superfamily; IHOG: Interference hedgehog; L3: third instar; MF: morphogenetic furrow; PTC: Patched; RT-PCR: reverse transcriptase PCR; SHH: Sonic hedgehog; SMO: Smoothened.

## Competing interests

The authors declare that they have no competing interests.

## Authors' contributions

DC performed the experiments, participated in their design, analysis and interpretation, and drafted the manuscript. KC participated in the genetic screen, AL constructed the UAS-ihog transgene, and both helped in data analysis and interpretation. DJvM and FC conceived of the study, and participated in its design and coordination and drafted the manuscript. All authors read and approved the final manuscript.

## Supplementary Material

Additional file 1 **Ectopic wing veins caused by overexpression of Boi**. **(A) **Wing from a EP1447/+; *ap*^*GAL4*^/+ fly, showing an ectopic vein (arrow and magnified boxed area) located between veins L3 and L4. Additional wing defects were also observed, though these were variable (arrowheads). **(B) **Wing from a fly of the genotype EP1447, *boi*^*C1*^/+; *ap*^*GAL4*^/+, showing complete suppression of the ectopic vein defect. Click here for file
