# Supplementary figures and images for: Ihog and Boi are essential for Hedgehog signaling in Drosophila
Source: Neural Dev. 2010 Nov 2;5:28. doi: 10.1186/1749-8104-5-28 (PMC2984377; doi:10.1186/1749-8104-5-28)

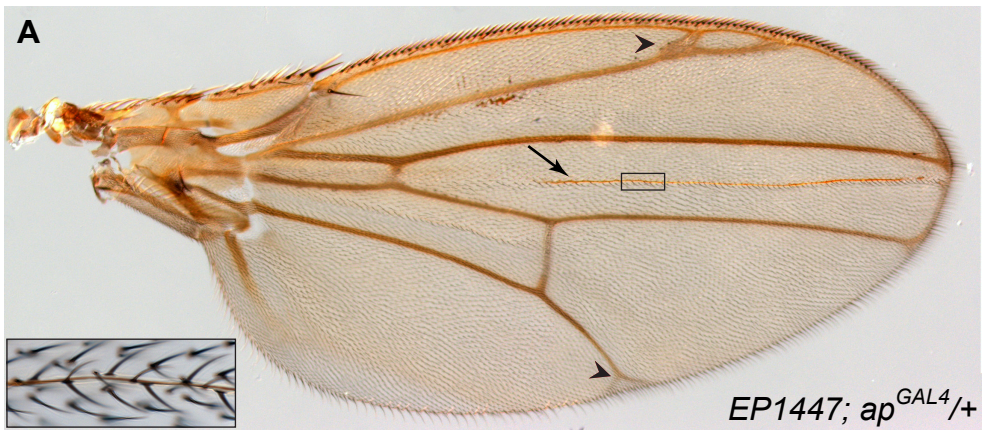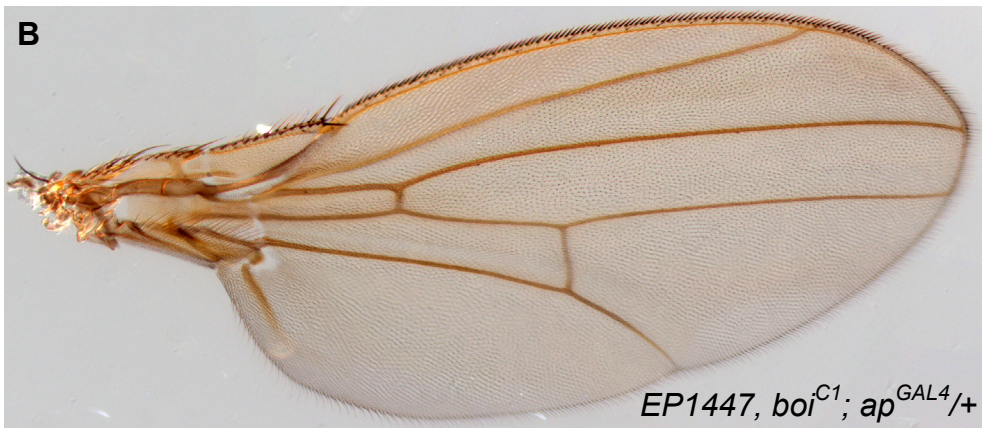

Supplement: Additional file 1 — Ectopic wing veins caused by overexpression of Boi. (A) Wing from a EP1447/+; apGAL4/+ fly, showing an ectopic vein (arrow and magnified boxed area) located between veins L3 and L4. Additional wing defects were also observed, though these were variable (arrowheads). (B) Wing from a fly of the genotype EP1447, boiC1/+; apGAL4/+, showing complete suppression of the ectopic vein defect. [file 1749-8104-5-28-S1.PDF]
